# Vitamins and periodontitis

**DOI:** 10.4103/0975-7406.76503

**Published:** 2011

**Authors:** Rajiv Saini

**Affiliations:** Department of Periodontology and Oral Implantology, Rural Dental College, Loni, Tehsil - Rahata, Ahmednagar - 413 736, Maharashtra, India. E-mail: drperiodontist@yahoo.co.in

Sir,

Periodontitis is a destructive inflammatory disease of the supporting tissues of the teeth and is caused either by specific microorganisms or by a group of specific microorganisms, resulting in progressive destruction of periodontal ligament and alveolar bone with periodontal pocket formation, gingival recession, or both.[1] Diet and nutrition impact on many oral diseases, in particular gingival and periodontal diseases. A person’s diet can exert a topical or a systemic effect on the body and its tissues. Before tooth eruption, foods provide a nutritional or systemic effect during tooth development and in the maturation of dentine and enamel. After the tooth erupts, foods play a topical or dietary role in the maintenance of tooth structure. It is well known that the caries process can be modified through dietary (food selection and eating habit changes) rather than nutritional changes.[2] Vitamins are organic compounds required as nutrients in tiny amounts by the organism and are required for the body to maintain appropriate metabolic reactions. Vitamins can be grouped as fat-soluble or water-soluble. By convention, the term vitamin does not include other essential nutrients such as dietary minerals, essential fatty acids, or essential amino acids (which are needed in larger amounts than vitamins). Vitamins have diverse biochemical functions. Some have hormone-like functions as regulators of mineral metabolism, or regulators of cell and tissue growth and differentiation, and others function as antioxidants. A largest number of vitamins function as precursors for enzyme cofactors that help enzymes in their work as catalysts in metabolism. Thirteen vitamins are presently universally recognized and are classified by their biological and chemical activity. Vitamins A, D, E, and K are fat-soluble, whereas vitamins B and C are water-soluble. Water-soluble vitamins dissolve easily in water and in general are readily excreted from the body, so consistent daily intake is important. Fat-soluble vitamins are absorbed through the intestinal tract with the help of lipids (fats). Greater possibilities to accumulate in the body, fat-soluble vitamins are more likely to lead to hypervitaminosis than are water-soluble vitamins. The first signs of deficiency of some micronutrients are seen first in the mouth, such as glossitis, cheilitis, and gingivitis. Undernutrition exacerbates the severity of oral infections and is a contributing factor to life-threatening diseases such as noma. Periodontal disease is associated with an increased production of reactive oxygen species which, if not buffered sufficiently, cause damage to the host cells and tissues. Antioxidant nutrients, for example, ascorbic acid, beta-carotene, and alpha-tocopherol, are important buffers of reactive oxygen species and are found in many fruits, vegetables, grains, and seeds. Current research is investigating the potential protective role of antioxidant nutrients in periodontal disease, and the most prudent approach is to recommend a daily intake of fruits and vegetables as a likely source of essential nutrients.
